# Identifying pretreatment baseline factors predictive of distant metastasis in patients with nasopharyngeal carcinoma after radiotherapy

**DOI:** 10.1097/MD.0000000000006692

**Published:** 2017-04-28

**Authors:** Yu Wang, Guojian Chen

**Affiliations:** Department of Oncology, Jiangmen Central Hospital, Jiangmen, China.

**Keywords:** distant metastasis, nasopharyngeal carcinoma, predictive factor, survival

## Abstract

This retrospective study was performed to identify pretreatment baseline factors that could predict the development of distant metastasis (DM) in patients with nasopharyngeal carcinoma (NPC).

A cohort of 119 NPC patients undergoing radiotherapy (RT) or chemoradiotherapy (CRT) were recruited into the study. Among them, 51 developed DM (DM group) within 3 years after treatment and 68 did not (DM-free group). Various clinicopathological factors were measured before the treatment and analyzed by univariate as well as multivariate analyses for the potential correlation with DM development.

Univariate analysis revealed that increased peripheral lactate dehydrogenase (LDH) level, lower lymphocyte–monocyte ratio (LMR), higher neutrophil–lymphocyte ratio (NLR), advanced American Joint Committee on Cancer (AJCC) stage, advanced T stage, and advanced N stage were significantly correlated with the presence of DM. Multivariate analysis identified advanced AJCC stage and high LDH level were independent predictive factors for DM.

Routinely measured pretreatment clinical factors, including AJCC state and serum LDH level, could independently predict DM. These factors will benefit the selection of appropriate treatment options and improve the overall survival of NPC patients.

## 1 Introduction

Nasopharyngeal carcinoma (NPC) is a major health concern in Southeast Asia, particularly in southern China. In 2010, 41,503 patients were diagnosed with NPC and 20,058 died from the disease in China, corresponding to an incidence and a mortality of 3.16 of 100,000 and 1.53 of 100,000, and accounting for 1.34% of all new cancer cases and 1.03% of all cancer-related deaths, respectively.^[1]^ At the initial diagnosis, most NPC patients presented no clinical evidence of metastases and are frequently treated with radiotherapy (RT) alone or chemoradiotherapy (CRT).^[2]^ When 2-dimensional RT (2D-RT) was the predominant option for NPC, locoregional recurrence and distant metastasis (DM) represented 2 equally important causes of treatment failures.^[3–6]^ The later development and application of intensity-modulated radiotherapy (IMRT) has significantly reduced the locoregional recurrence, leaving DM responsible for 68.4% to 73.6% of all treatment failures among NPC patients.^[7–11]^ Therefore, it is essential to identify novel factors predictive of DM among NPC patients to enable early interference with more aggressive treatment options and to improve the overall patient survival.

Several molecular markers have shown values in predicting the survival and DM among NPC patients, yet technical challenges and high costs associated with detecting these markers generally preclude their use in clinic.^[10,12,13]^ Although other risk factors also influence survival,^[3,4,9,14,15]^ few studies have assessed the pretreatment baseline parameters to DM. These factors could play a significant role in the choice of treatment NPC patients upon initial diagnosis. To address this issue, we retrospectively analyzed the correlations between various pretreatment baseline factors and the development of DM among NPC patients. Specifically, we focused on DM within the first 3 years after initial RT or CRT treatment, since 77.0% to 82.4% of metastases develop within this timeframe.^[3,4,16]^

## 2 Materials and methods

### 2.1 Patients

This retrospective study was approved by the Institutional Review Board of the Jiangmen Central Hospital (Jiangmen, China). A cohort of 119 NPC patients between 18 and 79 years of age and admitted into the Jiangmen Central Hospital from January 2009 to August 2011 were recruited into this study. The inclusion criteria included: histologically confirmed NPC without evidence of DM before treatment; an Eastern Cooperative Oncology Group performance status of ≤2; adequate renal, cardiac, and liver function; and achievement of complete remission following either RT or CRT. Patients with missing data, clinical signs of sepsis or other inflammatory diseases, serious concurrent medical issues, and a history of other malignancies were excluded from this study.

### 2.2 Collection of pretreatment baseline parameters

The following clinicopathological information was collected from each patient before the initiation of any treatment: sex, age, pathological type, NPC stage as defined by the 7th edition of the American Joint Committee on Cancer (AJCC) staging system, blood test results, and treatment strategies including RT dose and type of chemotherapy. Pretreatment albumin (ALB) and lactate dehydrogenase (LDH) level were measured using a Hitachi-7080 automated chemistry analyzer (Hitachi, Japan), and white blood cell differential counts using an AC.T 5diff AL hematology analyzer (Beckman Coulter, USA). The peripheral neutrophil–lymphocyte ratio (NLR) was calculated as the ratio of absolute counts between the peripheral neutrophil and lymphocyte measurements. Finally, the peripheral lymphocyte–monocyte ratio (LMR) was calculated as the lymphocyte count divided by the monocyte count.

### 2.3 Treatment

ALL patients received RT, including 79 treated with conventional 2D-RT and 40 with 3-dimensional conformal RT (3D-CRT). The total doses delivered were 68 to 70 Gy to the gross tumor, 60 to 62 Gy to the involved areas of the neck, and 50 Gy to uninvolved areas. Boost irradiation not exceeding 6 Gy to the skull base and primary nodal sites was administered to patients presenting severe enlargement of the primary lymph nodes or showing no dissipation of lymph nodes after initial RT.

Chemotherapy was administered to 76 patients as a concurrent (n = 31), neoadjuvant (n = 13), adjuvant (n = 7), or a combination of 2 or all 3 options (n = 25), in addition to RT. The regimens for concurrent chemoradiotherapy (CCRT) were cisplatin alone, consisting of 30 to 40 mg/m^2^ cisplatin every week for 3 to 6 cycles during RT. For neoadjuvant or adjuvant chemotherapy, cisplatin or carboplatin plus one of the following 3 agents were used: 5-fluorouracil (5-FU), docetaxel (DOC), or gemcitabine. The doses were as follows: 20 to 25 mg/m^2^/day on days 1 to 4 for cisplatin; 300 to 400 mg/m^2^ on day 1 for carboplatin; 500 to 1000 mg/m^2^/day on days 1 to 5 for 5-FU; 75 mg/m^2^/day once every 3 to 4 weeks for 1 to 3 cycles for DOC; and 1 g/m^2^/day on day 1 and day 14 for gemcitabine. The exact doses were selected based on the patients’ conditions, and if severe side effects were experienced, the dose was reduced accordingly.

### 2.4 Follow-up

All patients were assessed every 3 to 6 months from the last date of RT, up to 3 years or the time when DM was detected, whichever occurred first. DM was diagnosed based on clinical symptoms, physical examination and imaging, including chest radiography, bone scan, computed tomography, and abdominal ultrasonography.

### 2.5 Statistical analysis

Statistical analysis was performed using SPSS software (version 19.0). Qualitative variables were presented as frequencies and percentages, while quantitative variables as mean ± standard error (SE). Kaplan–Meier analysis was performed to assess the incidence of DM over time after the treatment. Univariate analysis was performed using Log-rank test. Parameters showing significance from the univariate analysis were used in multivariate analysis using a Cox proportional hazards regression model. A *P*-value of <.05 was considered statistically significant.

## 3 Results

In this study, we retrospectively analyzed 119 NPC patients with a mean age of 48 years, including 86 males and 33 females. Of them, 99.2% were diagnosed with nonkeratinizing NPC and 0.8% with adenocarcinoma. The general clinicopathological information of all patients are summarized in Table 1. Upon the diagnosis of NPC, 43 patients received RT alone and 76 went through CRT, including 31 (40.8%) receiving CCRT, 13 (17.1%) receiving neoadjuvant CRT only, 7 (9.2%) receiving adjuvant CRT only, and 25 (32.9%) receiving a combination of therapies (neoadjuvant plus adjuvant CRT, neoadjuvant plus CCRT, CCRT plus adjuvant CRT or neoadjuvant plus CCRT plus adjuvant CRT). Kaplan–Meier analysis showed that the incidence of DM increased over time following the treatment: from 16.81 ± 3.43% within the first 12 months, to 35.29 ± 4.38% within the first 2 years and 42.86 ± 4.54% within the first 3 years. On average, DM occurred at 26.97 ± 1.07 months after the initial RT or CRT treatment.

**Table 1:**
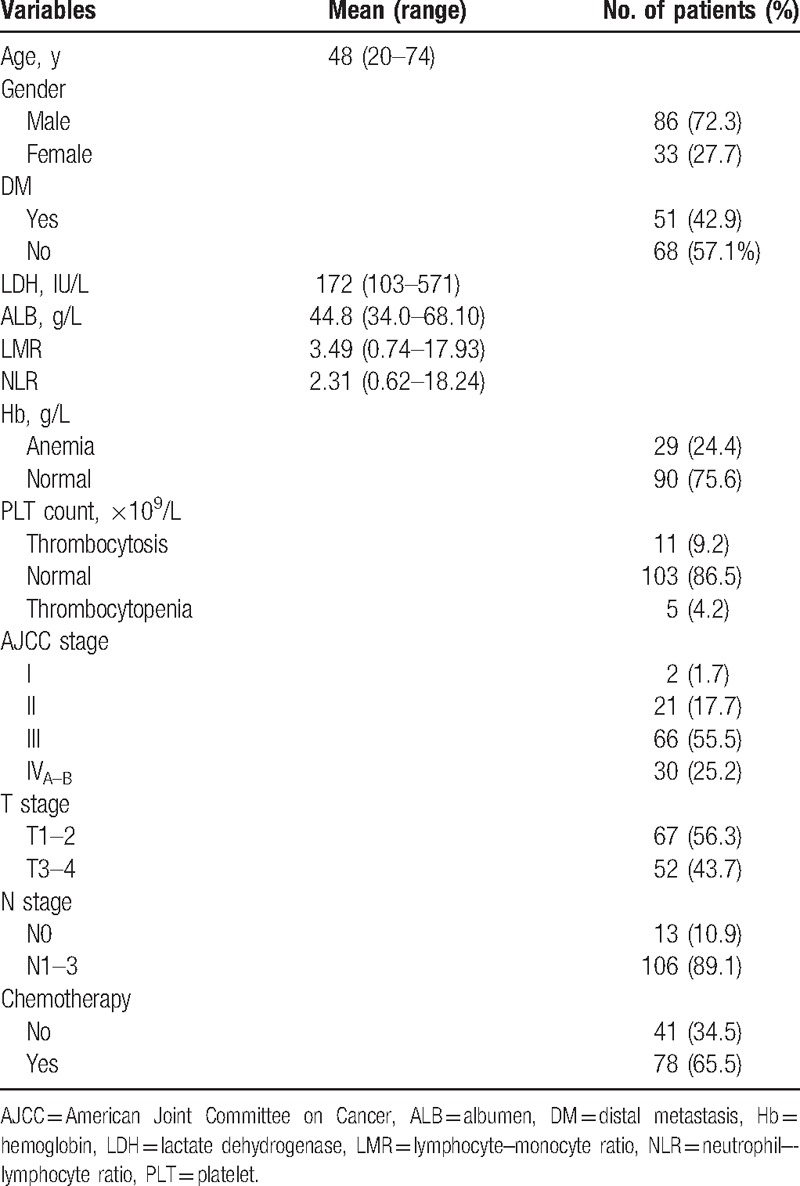
Baseline clinicopathological characteristics of all patients.

Univariate analysis (Table 2) revealed that increased LDH level (LDH >240 IU/L, *χ*^2^ = 15.911, *P* < .001), lower LMR value (LMR ≤ 3.49, *χ*^2^ = 5.166, *P* = .023), higher NLR value (NLR ≥ 2.60, *χ*^2^ = 4.126, *P* = .042), advanced AJCC stage (III/IV stage, *χ*^2^ = 8.776, *P* = .003), advanced tumor (T) stage (T3–4 stage, *χ*^2^ = 6.165, *P* = .013) and advanced lymph node (N) stage (N1–3 stage, *χ*^2^ = 6.116, *P* = .013) were significantly correlated with the presence of DM. In contrast, no significant correlations were noted between DM and gender (*χ*^2^ = 2.805, *P* = .094), age (*χ*^2^ = 3.584, *P* = .058) or ALB level (*χ*^2^ = 3.619, *P* = .057). The presence of anemia (*χ*^2^ = 2.252, *P* = .133) and thrombocytosis (*χ*^2^ = 0.077, *P* = .781) did not increase the risk of DM. Moreover, treatment with RT or CRT did not significantly affect the incidence of DM (*χ*^2^ = 0.166, *P* = .684).

**Table 2:**
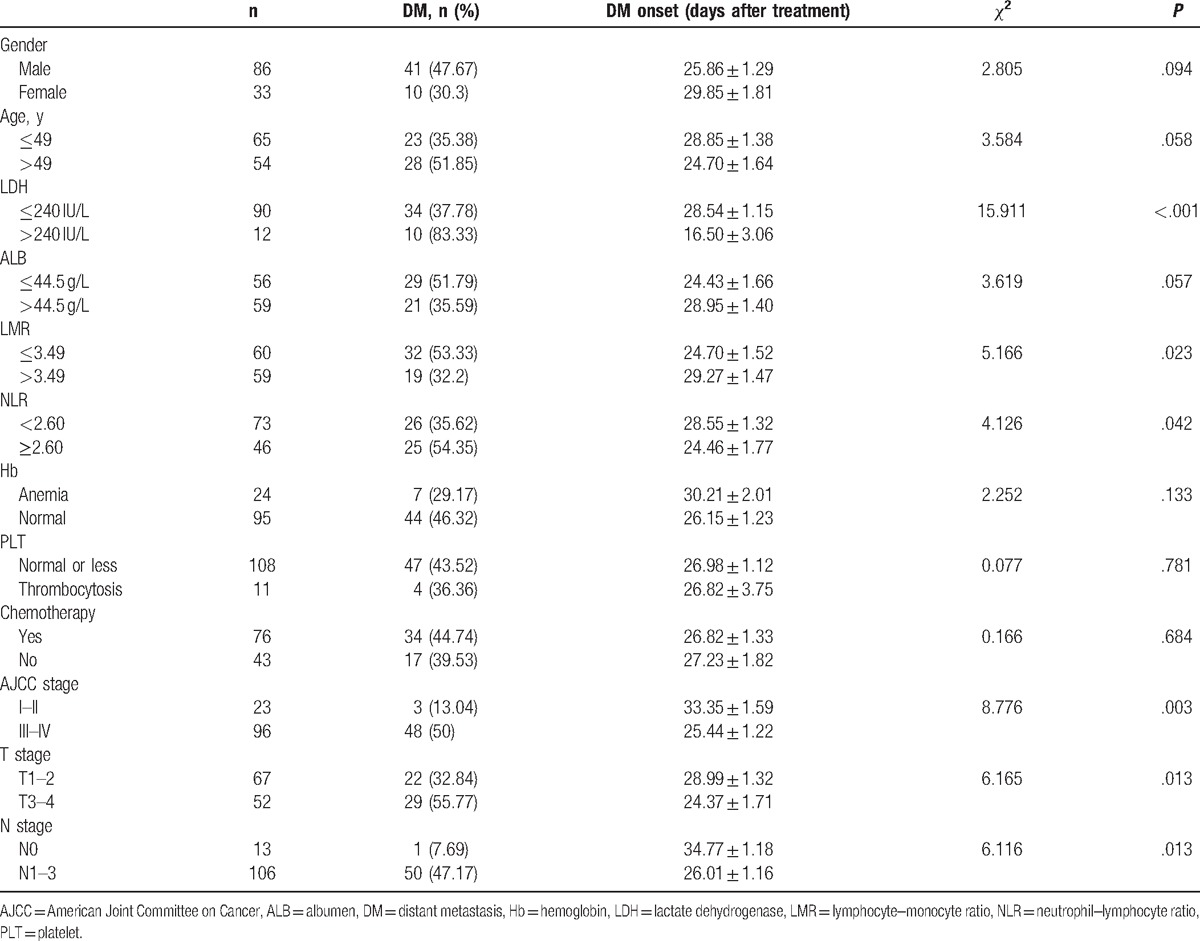
Univariate analysis using Log-rank test to identify potential risk factors for distant metastasis development (n = 119).

The pretreatment factors showing a *P* < .05 from univariate analysis were used in the multivariate analysis, which showed that only AJCC stage and LDH were independent risk factors of DM. An AJCC stage III–IV or high LDH level (>240 IU/L) significantly increased the risk of DM, with OR values of 5.043 (95% CI: 1.218–20.882; *P* = .026), and 3.420 (95% CI: 1.655–7.067; *P* = .001), respectively (Table 3).

**Table 3:**
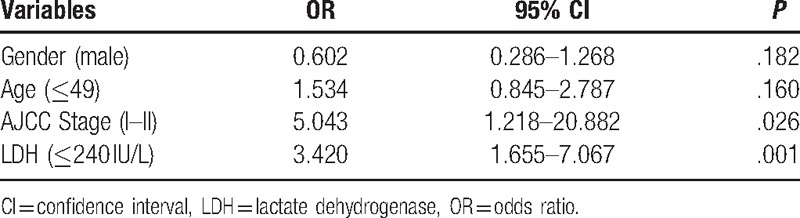
Multivariate analysis using a Cox proportional hazards regression model.

## 4 Discussion

Survival in NPC is severely compromised by the development of DM, whether in the early 2D-RT period or following treatment with IMRT,^[3–9,11]^ indicating that DM is a formidable task for the treatment of NPC.^[17,18]^ Patients without obvious clinical evidence of DM at the time of initial diagnosis may already have subclinical micrometastases not detected by routine examinations.^[19,20]^ This may explain why the majority of DM develops within 3 years of treatment. Thus, the key strategy to improve the survival of NPC patients is to be able to predict the risk of DM and to select the treatment options accordingly. In this study, we retrospectively analyzed the correlations between various pretreatment baseline factors and DM to identify those predictive of DM and thus high-risk patients who may benefit from early aggressive therapy.

We first used the Log-rank test for univariate analysis to identify pretreatment clinical factors significantly correlated with DM after RT. Given that many clinical factors are interrelated, it is challenging to separate factors or to identify relationships between factors. Therefore, we included factors showing a *P* < .05 from the univariate analysis in the multivariate analysis, and established an optimal formula to accurately predict DM.

We found that an advanced clinical stage (AJCC III/IV) at the time of diagnosis had a strong impact on predicting DM, consistent with previous reports.^[3,4,9,21]^The AJCC N stage and AJCC T stage, although significant factors for DM by univariate analysis, were not significant by multivariate analysis. The value of AJCC T stage for predicting DM varied between studies.^[4,9,21]^

We found that the pretreatment baseline LDH level significantly correlated with DM development. Patients with higher LDH were more likely to develop DM, as reported previously.^[2,3,22]^ In spite of extensive efforts, the underlying mechanisms linking LDH to DM remain largely unknown,^[23–25]^ although it was noted that elevated serum LDH levels were associated with advanced clinical stage.^[22]^

In addition, the pretreatment LMR value and pretreatment NLR were sometimes used to study the prognosis of NPC. A meta-analysis revealed that enhanced LMR was significantly associated with favorable overall survival in patients with digestive system cancers (HR = 0.63, 95% CI: 0.49–0.81), urinary tract tumors (HR = 0.66, 95% CI: 0.52–0.84), lung cancer (HR = 0.62, 95% CI: 0.54–0.72), and NPC (HR = 0.50, 95% CI: 0.43–0.57).^[26]^ This is likely because inflammation has been confirmed as a key component of cancer progression, and lymphocytes and monocytes are important inflammatory components. Pretreatment LMR reflects the balance of lymphocytes and monocytes.^[27,28]^ We also found a few studies have examined the relationship between NLR and DM in cancer patients.^[22,29,30]^ One found that NLR > 5 predicts shorter overall survival in patients with head and neck squamous cell carcinoma.^[29]^ Another study demonstrated that NLR > 2.81 was a significant adverse independent predictive factor for DM.^[30]^ However, in our study, the pretreatment LMR value and pretreatment NLR were not statistically significant by multivariate analysis, although its *P*-value was <.05 by univariate analysis. The relationship between these 2 factors and metastasis of NPC remains to be further explored.

Other factors, including age, sex, pretreatment platelet count, serum ALB level, and anemia were not significantly correlated with DM in this study, which is consistent with other studies.^[8,21,22,31–34]^

It is noteworthy that no significant difference in DM was observed between patients receiving RT and those receiving CRT. The CRT options used in this study included concurrent, neoadjuvant, and adjuvant chemotherapy. However, it is controversial which treatment is more effective. Although it is commonly agreed that concurrent cisplatin-based CRT is the standard treatment plan for locally advanced NPC,^[35,36]^ our study showed no advantage for any treatment option. However, the limited number of patients receiving each treatment in this study may obscure the potential significance of any regimen. In addition, analyzing patients from a single center may also generate selection bias. Therefore, future studies involving a greater number of patients from multiple centers and receiving different treatments are needed to the impacts of treatment options on DM and patient survival.

In summary, we identified that advanced AJCC stage and high LDH level were independent, significant risk factors for DM in NPC patients. As routinely measured factors before the initiation of treatment, they represent easily accessible, inexpensive, and robust predictors to be used in clinic. The findings from this study will pave the way for improved prediction of patient prognosis and thus a better selection of treatment options for NPC patients.
